# Efficacy of Ultrasound-Guided Serratus Anterior Plane Block for Managing Pain Due to Multiple Rib Fractures: A Scoping Review

**DOI:** 10.7759/cureus.21322

**Published:** 2022-01-17

**Authors:** Abhijit Nair, Sandeep Diwan

**Affiliations:** 1 Anaesthesiology, Ibra Hospital, Ibra, OMN; 2 Anaesthesiology, Sancheti Institute for Orthopaedics and Rehabilitation, Pune, IND

**Keywords:** ultrasound-guided, serratus anterior plane block, multiple rib fractures, ultrasound guided regional anesthesia, nerve block, acute pain management

## Abstract

Ultrasound (US) guided serratus anterior plane block (SAPB) is a fascial plane block that has been utilized for managing pain after thoracotomy, mastectomy, and fractured ribs. We conducted this qualitative review to investigate the analgesic efficacy of US-guided SAPB in patients who sustained multiple rib fractures (MRFs).

We registered our review proposal in a prospective register of systematic reviews, PROSPERO, with identifier CRD42020177145. This review adheres to Preferred Reporting Items for Systematic Reviews and Meta-Analyses (PRISMA) guidelines for the identification, screening, and inclusion of relevant articles. Two authors independently searched Pubmed, Embase, the Cochrane Library, Google Scholar, and Web of Science to identify available randomized controlled trials (RCT), case reports, case series reports where SAPB was used for managing pain due to MRFs.

Out of the 66 articles identified by the search strategy, 23 articles were assessed for eligibility, and 16 articles were included in the qualitative review. Due to significant heterogenicity, the presence of only one RCT, the presence of case report or series, availability of only retrospective studies for review, a quantitative analysis using statistical tests were not done. Grading of Recommendations Assessment, Development, and Evaluation (GRADE) assessment was not done as there was only one RCT in the review which had limitations like allocation concealment and blinding.

US-guided SAPB is a safe and effective fascial plane block for managing pain in patients who sustain MRFs. Further research in the form of well-designed and adequately powered RCTs is needed to confirm its use in patients with MRFs.

## Introduction and background

Patients sustain multiple rib fractures (MRFs) due to various etiologies like road traffic accidents, assault, falls from heights, etc. The morbidity and mortality increase significantly in presence of other injuries, and in elderly patients with comorbidities [1,2]. Pulmonary complications like pneumonia, flail chest, pneumothorax, hemothorax, acute lung injury requiring non-invasive or invasive ventilation contributes to morbidity, prolonged hospital stay, and thus increased cost of treatment. Poorly controlled pain leads to basal atelectasis, worsening of acute lung injury, non-invasive or invasive ventilation, prolonged hospital stays, and thus an overall increased burden of the cost of treatment [3,4]. Pain management offered for patients with MRFs could be either systemic analgesia (opioids, multimodal analgesia with adjuvants) or regional anesthesia (RA). There are several RA options that can be offered to alleviate pain following MRFs like thoracic epidural analgesia, paravertebral block, intercostal nerve block, serratus anterior plane block (SAPB), or erector spinae plane block [5-10].

**Figure 1:**
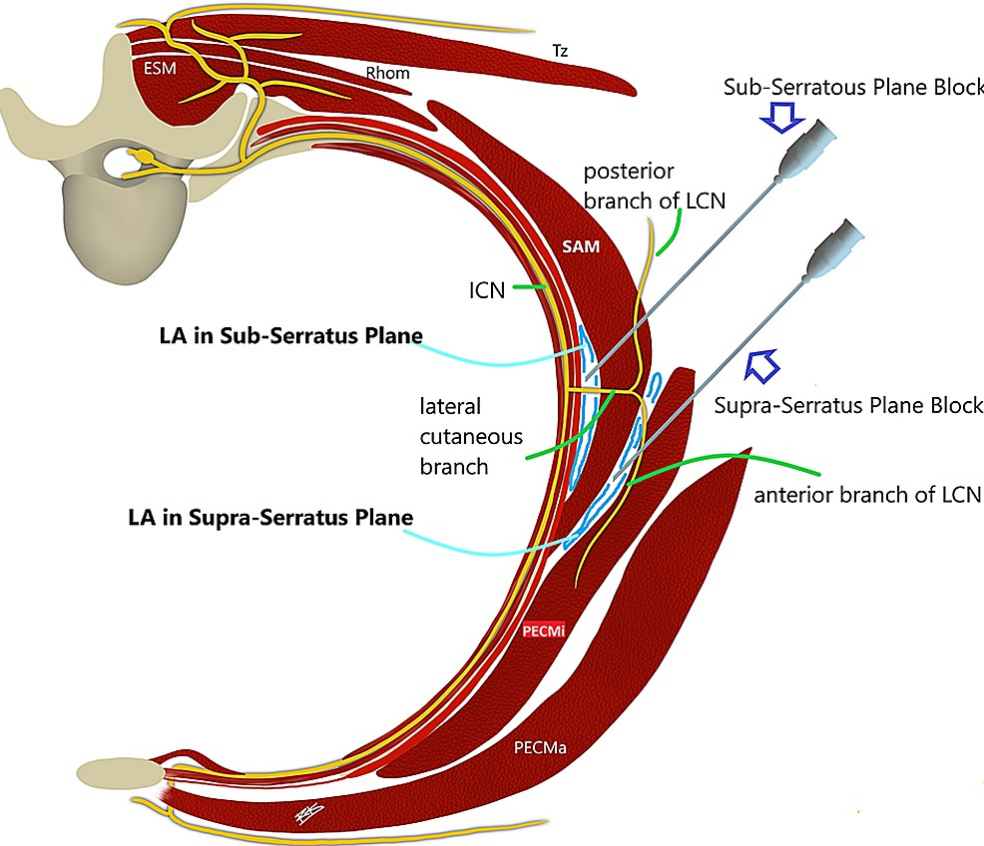
Schematic diagram showing needle placement for ultrasound-guided serratus anterior plane block, in superficial and deep plane Abbreviations: LA - local anesthetic, ICN - intercostal nerve, LCN - lateral cutaneous nerve, Rhom - rhomboids muscle, ESM - erector spinae muscle, Tz - trapezius muscle, SAM - serratus anterior muscle, PECMa - pectoralis major muscle, PECMi - pectoralis minor muscle

**Figure 2:**
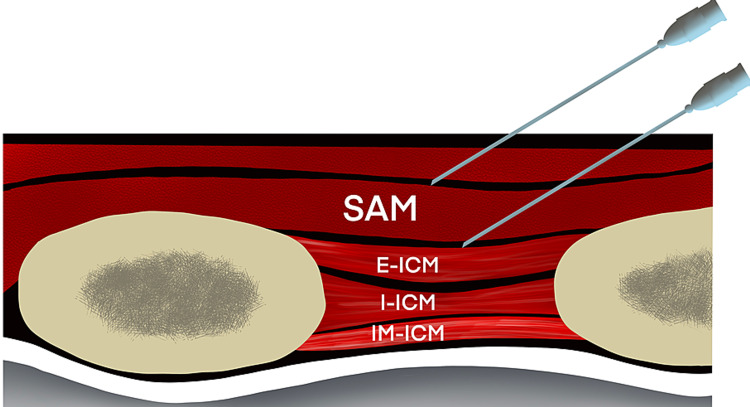
Schematic diagram showing needle placement for superficial and deep serratus anterior plane block along with various relevant structures Abbreviations: R - Rib, LD - latissimus dorsi muscle, E-ICM - external intercostal muscle, I-ICM - internal intercostal muscle, IM-ICM - innermost intercostal muscle, SAM - serratus anterior muscle

Ultrasound (US) guided SAPB was initially described by Blanco et al. in 2013. Blanco et al. described two planes, one superficial to serratus anterior muscle and second underneath the muscle and above the rib (Figures 1, 2) [11,12]. When a SAPB is performed, it targets the lateral cutaneous branches of the thoracic intercostal nerves arising from the ventral rami of the thoracic spinal nerves. These nerves traverse through the internal intercostal, external intercostal, and SA muscles to innervate the muscles of the anterolateral aspect thoracic cage. These branches travel through the two potential spaces above and below the SA muscle. At the level of the fifth rib, the superficial plane is defined as the fascial plane formed by the anterior aspect of the SA muscle and the posterior aspect of the latissimus dorsi muscle. The deep plane of the fascial plane is the plane between the posterior aspect of the SA muscle and the external intercostal muscles and ribs. LA injected in either of these planes spreads throughout the lateral chest wall along these fascial planes and thereby providing analgesia from T2-T9 dermatomes of the anterolateral thorax. Due to the ease of identification of relevant structures using the US, the block was extensively utilized for managing postoperative pain after breast surgeries, thoracoscopic non-cardiac surgeries, minimally invasive cardiac surgeries, thoracotomy, and chest trauma including rib fractures, especially at the posterolateral aspect.

Most of the published articles are case reports, case series, or retrospective data. Although the case reports and series mention the efficacy of US-guided SAPB in patients who sustain MRFs, there is a lacuna in the existing literature in the form of well-designed randomized controlled trials (RCTs). We conducted a qualitative review of the studies to examine the effectiveness of US-guided SAPB in patients who sustained MRFs, which is our primary outcome. The secondary outcomes are pain scores and complications due to US-guided SAPB if any.

## Review

Methodology

We registered our review proposal in a prospective register of systematic reviews, PROSPERO, with identifier CRD42020177145. We performed this review to examine the effectiveness of US-guided SAPB in patients who sustained MRFs. This review adheres to Preferred Reporting Items for Systematic Reviews and Meta-Analyses (PRISMA) guidelines for the identification, screening, and inclusion of relevant articles [13]. The period of the review was from June 2020 to June 2021.

Search methods for identification of studies

A collection of studies was conducted by AN and SD. The manuscripts meeting the inclusion criteria were assessed, and data were extracted following a standardized format by the same authors. Pubmed, Embase, the Cochrane Library, Google Scholar, and Web of Science were searched to identify available RCTs, case reports, case series reports, and use of SAPB for rib fractures without any language restriction. The search strategy for Pubmed was: (serratus anterior plane block [All Fields] AND rib fractures [All Fields]) OR multiple rib fractures [All Fields] AND "rib fractures"[MeSH Terms]. We manually retrieved and analyzed all the articles generated by the above search strategy. We also searched for conference abstracts, posters, thesis with the above-mentioned keywords.

Selection criteria and data extraction

**Figure 3:**
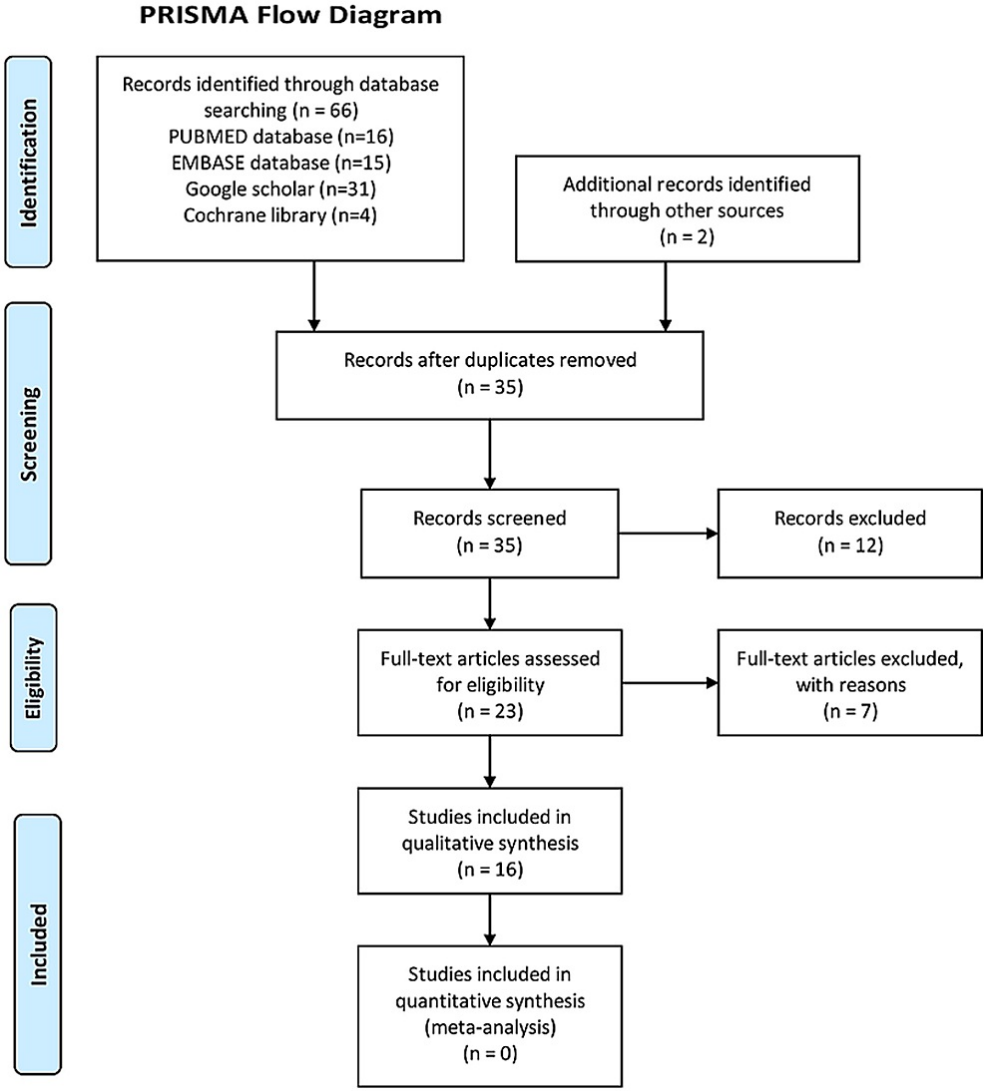
PRISMA flow diagram depicting included and excluded studies

We used the patients, interventions, comparisons, and outcomes (PICO) format to identify components of clinical evidence. Studies that were to be included were: 1) patients with rib fractures, 2) intervention: US-guided SAPB for pain relief, 3) comparison: no intervention or multimodal analgesia, 4) outcomes: pain scores and opioid consumption. Patient age was not an exclusion criterion. Exclusion criteria were multiple injuries including head injuries, visceral and long bone fractures, intubated patients, duplicate publications, cadaveric studies patients undergoing surgery after MRFs. All titles and abstracts were meticulously scanned for eligibility. Thereafter the full text was reviewed to ensure if the paper fulfills the criteria laid above for inclusion. Figure 3 (PRISMA flow chart) depicts the process of selection of papers for review.

Data synthesis

We identified 66 articles which was a mix of case reports, series, retrospective studies, and one RCT using the above-mentioned keywords. Data relevant to the outcomes of interest were extracted from each study. After removing duplicate articles, after assessing articles that were considered eligible based on inclusion criteria, and excluding articles that were not from peer-reviewed journals, we included 16 articles in the qualitative review.

From all the published articles finally selected, the following information was gathered and entered in tables: age, gender, LA used (concentration, volume, drug), single-shot injection or continuous infusion, if continuous infusion for how many hours/days and volume with the concentration of LA used, pain scores monitored, rescue analgesic used (a drug used, number of doses, total rescue analgesic used), patient satisfaction scores and complications.

Result analysis

Search Results

Using the search strategy mentioned above in methods, we identified 66 relevant articles. After removing 31 duplicate articles, we screened 35 suitable articles for eligibility. Another 12 articles were excluded as the end-points were heterogeneous or involved some other intervention along with SAPB. Finally, 23 articles were assessed for eligibility and 16 articles were included in the qualitative review. Due to significant heterogenicity, the presence of only one RCT, having only case reports or series, only retrospective studies for review, a quantitative analysis using statistical tests was not done.

Study Characteristics

The only RCT was the one by Tekşen et al. in which the authors randomized 60 patients into two groups: in one group single US-guided SAPB was performed using 30 mL of 0.25% bupivacaine and the control group was on PCA tramadol [14]. Patients were monitored for pain scores over 24 hours and pain scores were compared. The mean score was 1 in the SAPB group, and 2.7 in the control group. Patient satisfaction scores were not documented. Lack of blinding, allocation concealment, and heterogenicity were limitations of this RCT. In a series of 10 patients, Paul et al. performed a single US-guided SAPB in patients with three or more unilateral rib fractures having pain scores of about 9/10 on arrival. They injected up to 40 mL of 0.25% ropivacaine depending upon the body weight of the patient. The mean pain score at 30 min was 4 and at 60 min was 2.1. There were no block-related complications [15]. Schnekenburger et al. conducted a pilot study in 20 patients with MRFs by performing single-shot US-guided SAPB. Mean scores at baseline and 4 hrs were 6.5 (6-8) and 3 (2-5) [16]. In another retrospective study by Diwan et al. involving 72 patients out of which 38 patients received continuous SAP infusion via an indwelling catheter. The authors retrospectively compared analgesic efficacy and 24 hr fentanyl consumption of continuous SAPB with fentanyl infusion [17]. On analysis, authors found that there were statistically significant lower pain scores in patients of SAPB group when compared to that of fentanyl and in also in 24 hrs fentanyl consumption in patients who received continuous SAPB versus that in fentanyl group (p=0.001). However, the study was retrospective and had a small sample size. There was also significant heterogenicity in terms of age and other associated injuries. There were several case reports and series which were identified on literature search. Papers in which SAPB was performed in critically ill patients with multiple injuries were not analyzed as they did not fulfill the inclusion criteria [18,19].

Risk of Bias in Included Studies

There was only one RCT that was available for review which had several limitations. There was no random sequence generation or allocation concealment done which led to selection bias. Blinding of participants and personnel is important to avoid performance and detection bias which was not possible in the selected studies [20]. Attrition and reporting bias is due to incomplete reporting data and selective reporting, respectively [21]. As the data analyzed in this review was mostly from case reports and series, there was no attrition data or reporting bias as such. As fewer studies were included, the funnel plot was not evaluated for publication bias. A quantitative analysis of data or meta-analysis was not performed due to limited sample size, heterogenicity, and reporting bias. Quantitative analysis of non-comparative case series does not produce relative association measures such as odd’s ratio or relative risks. There was only one RCT in the review, which had limitations like allocation concealment and blinding. Therefore, it was not performed in this review. For this reason, the Grading of Recommendations Assessment, Development, and Evaluation (GRADE) assessment was not done.

Discussion

To date, no review article has evaluated the effectiveness and safety of SAPB in patients with MRFs. The results indicated that US-guided SAPB could significantly decrease the postoperative pain score and opioids requirement and is safe without significant adverse events. There were case reports and case series and only one RCT for analysis. The quality of the evidence was very low.

Biswas et al. demonstrated in a cadaveric study that SAP performed superficial or deep did not influence the spread of injectate either in anteroposterior or craniocaudal direction [22]. Being a fascial plane block, a higher volume of LA (around 30 mL or more) is expected to provide a better quality of analgesia [23,24]. A volume of 30-40 mL LA is required to achieve sensory loss from dermatome T2-T9 in SAPB [25]. On reviewing the results, we observed that patients with MRFs who received either a single shot or continuous SAPB have better pain scores from baseline. However, the absence of a control group was a big limitation. Therefore, we could not conclude if pain scores with intervention and pain scores with analgesics like opioids or any combination of multimodal analgesia would be comparable or better. Patient satisfaction scores were also not consistently mentioned in the papers published. The GRADE of evidence could not be performed in our review due to several reasons. Most of the articles included were case reports or case series due to which the sample size was very small. There was no standardized way of reporting pain scores, LA volume/concentration was inconsistent thus leading to significant heterogenicity. We agree with the fact that a review of case reports or case series cannot be placed at the top of the hierarchy in a pyramid that depicts validity [26]. It is not possible to randomize patients with fracture ribs into different groups due to several reasons. The rib fractures are not always unilateral. In situations when patients present with unilateral MRFs, there is a possibility that there could be other injuries as well. A group of patients will require surgery for abdominal/head/long bones trauma and thus will be excluded from intervention. Lastly, there can be ethical concerns in randomizing patients with multiple injuries. This explains the dearth of RCTs included in this review and thus the limited sample size. The details of case reports are depicted in Table 1.

**Table 1:** Details of case reports, series, and various studies in which US-SAPB was used in patients with multiple rib fractures

Study/ year	No. of patients	Local anesthetic, dose, and volume	Characteristics	Pain scores
Tekşen et al., 2020 [14]	RCT with 30 patients in each group Group 1- SAPB Group B- tramadol PCA	30 mL of 0.25% bupivacaine	Single-shot injections	Mean score over 24 hrs: 1 in SAPB group, 2.7 in control group
Paul et al., 2020 [15]	Series of 10 patients	Up to 40 mL of 0.25% bupivacaine	Single-shot injections	Mean score at 30 min: 4.4 Mean score at 60 min: 2.1
Schnekenburger et al., 2021 [16]	Pilot study of 20 patients	30 mL of 0.5% ropivacaine	Single shot	Mean pain score: Baseline-6.5(6-8) 4 hrs-3 (2-5)
Diwan et al., 2021 [17]	Retrospective study, comparison with fentanyl infusion: 3 patients received SAPB	25 mL of 0.2% ropivacaine with 50 μg fentanyl	0.1% ropivacaine – 8 mL/hr	Mean score: 1-3
Camacho et al., 2018 [27]	1, 33 yr/M	20 mL of 0.25% levobupivacaine	Continuous infusion: 0.12% levobupivacaine @ 5 mL/hr for 5 days	0-3 No rescue analgesia
Kunhabdulla et al., 2014 [28]	1, 63 yr/M	20 mL of 0.125% bupivacaine	Continuous infusion of 20 mL of 0.0625% bupivacaine with 1 μg/mL fentanyl for 6 days	No rescue analgesic
Bossolasco et al., 2017 [29]	1, 63/M	30 mL of LA (15 mL of ropivacaine 0.125% + 15 mL of lignocaine 1%),	0.125% ropivacaine @ 5 mL/hr for 7 days	0-2 throughout
Lin et al., 2020 [30]	6 (Median 81.5 yrs)	1-30 mL of 0.25% bupivacaine 2-30 mL of 0.25% bupivacaine 3-20 mL 0.5% bupivacaine 4-30 mL of 0.25% bupivacaine 5- 30 mL of 0.25% bupivacaine 6. 20 mL of 0.25% bupivacaine	All were single shot injections	Significant pain relief in all patients (pain scores not mentioned)
Fu et al., 2016 [31]	1, 98 yr/F	40 mL 0.25% ropivacaine	0.2% bupivacaine @ 10 mLhr for 5 days	0-2
Rose et al., 2019 [32]	1, 39 yr/M	30 mL 0.5% ropivacaine	0.2% ropivacaine @ 5 mL/hr for 7 days	0-2
Durant et al., 2016 [33]	2 patients: 82 hr male, 65 yr female	30 mL of 0.5% ropivacaine	Single shot	Patient 1-8/10 before and 0/10 30 min after block. Patient 2-9/10 prior and 2/10 later
Hernandez et al., 2019 [34]	Retrospective study, 34 patients	Inconsistent LA- Varying concentration and volumes of bupivacaine, ropivacaine	12 mL/hr of 0.2% ropivacaine	Baseline: 7 (6,9) After block: 3 (0,4)
Martel et al., 2020 [35]	27 patients	0.2% ropivacaine	8-14 mL/hr of 0.2% ropivacaine	Pain scores not mentioned
Martinez et al., 2019 [36]	10 patients	Up to 30 mL of 1% lidocaine	3 single shot, 7- continuous LA infusion-0.2% ropivacaine up to 12 ml/hr	Baseline: 7.3 [5.3–8.8] After block: 4 [3.6–4.6]
McLean et al., 2019 [37]	67 yr/M	40 mL of 0.375% ropivacaine	Single shot	Before block - 10/10 After block - 0/10
Rose et al., 2019 [38]	5 patients	20 mL 0.5% ropivacaine	0.2% ropivacaine at 5 mL/hr with 8 mL bolus on demand with 30 min lockout	Pain score: 8-9 before block, After block (from day 1): 0-4

The limitations of this review are the inadequate number of RCTs, small sample size, and absence of sub-group analysis due to a limited number of cases included in the review. A quantitative review was not performed due to the above-mentioned issues.

## Conclusions

US-guided SAPB appears to be a safe and effective fascial plane block for managing pain in patients who sustain MRFs. A continuous LA infusion with an indwelling catheter for 3-5 days is better when compared to the single-shot technique. Due to the small sample size and low quality of evidence, further studies with large sample size and high-quality researches are needed.

## References

[1] Sikander N, Ahmad T, Shaikh KA Sr, Abid A, Mazcuri M, Nasreen S (2020). Analysis of injury patterns and outcomes of blunt thoracic trauma in elderly patients. Cureus.

[2] Narayanan R, Kumar S, Gupta A (2018). An analysis of presentation, pattern and outcome of chest trauma patients at an Urban Level 1 Trauma Center. Indian J Surg.

[3] Dogrul BN, Kiliccalan I, Asci ES, Peker SC (2020). Blunt trauma related chest wall and pulmonary injuries: an overview. Chin J Traumatol.

[4] Chrysou K, Halat G, Hoksch B, Schmid RA, Kocher GJ (2017). Lessons from a large trauma center: impact of blunt chest trauma in polytrauma patients-still a relevant problem?. Scand J Trauma Resusc Emerg Med.

[5] El-Boghdadly K, Wiles MD (2019). Regional anaesthesia for rib fractures: too many choices, too little evidence. Anaesthesia.

[6] Womack J, Pearson JD, Walker IA, Stephens NM, Goodman BA (2019). Safety, complications and clinical outcome after ultrasound-guided paravertebral catheter insertion for rib fracture analgesia: a single-centre retrospective observational study. Anaesthesia.

[7] Adhikary SD, Liu WM, Fuller E, Cruz-Eng H, Chin KJ (2019). The effect of erector spinae plane block on respiratory and analgesic outcomes in multiple rib fractures: a retrospective cohort study. Anaesthesia.

[8] Yeying G, Liyong Y, Yuebo C, Yu Z, Guangao Y, Weihu M, Liujun Z (2017). Thoracic paravertebral block versus intravenous patient-controlled analgesia for pain treatment in patients with multiple rib fractures. J Int Med Res.

[9] Hwang EG, Lee Y (2014). Effectiveness of intercostal nerve block for management of pain in rib fracture patients. J Exerc Rehabil.

[10] Ekpe EE, Eyo C (2017). Effect of analgesia on the changes in respiratory parameters in blunt chest injury with multiple rib fractures. Ann Afr Med.

[11] Blanco R, Parras T, McDonnell JG, Prats-Galino A (2013). Serratus plane block: a novel ultrasound-guided thoracic wall nerve block. Anaesthesia.

[12] Southgate SJ, Herbst MK (2021). Ultrasound Guided Serratus Anterior Blocks. https://www.ncbi.nlm.nih.gov/books/NBK538476/.

[13] Moher D, Liberati A, Tetzlaff J, Altman DG (2009). Preferred reporting items for systematic reviews and meta-analyses: the PRISMA statement. PLoS Med.

[14] Tekşen Ş, Öksüz G, Öksüz H (2021). Analgesic efficacy of the serratus anterior plane block in rib fractures pain: a randomized controlled trial. Am J Emerg Med.

[15] Paul S, Bhoi SK, Sinha TP, Kumar G (2020). Ultrasound-guided serratus anterior plane block for rib fracture-associated pain management in emergency department. J Emerg Trauma Shock.

[16] Schnekenburger M, Mathew J, Fitzgerald M, Hendel S, Sekandarzad MW, Mitra B (2021). Regional anaesthesia for rib fractures: a pilot study of serratus anterior plane block. Emerg Med Australas.

[17] Diwan S, Nair A (2021). A retrospective study comparing analgesic efficacy of ultrasound-guided serratus anterior plane block versus intravenous fentanyl infusion in patients with multiple rib fractures. J Anaesthesiol Clin Pharmacol.

[18] Bhalla PI, Solomon S, Zhang R, Witt CE, Dagal A, Joffe AM (2021). Comparison of serratus anterior plane block with epidural and paravertebral block in critically ill trauma patients with multiple rib fractures. Trauma Surg Acute Care Open.

[19] Beard L, Hillermann C, Beard E, Millerchip S, Sachdeva R, Gao Smith F, Veenith T (2020). Multicenter longitudinal cross-sectional study comparing effectiveness of serratus anterior plane, paravertebral and thoracic epidural for the analgesia of multiple rib fractures. Reg Anesth Pain Med.

[20] Kabisch M, Ruckes C, Seibert-Grafe M, Blettner M (2011). Randomized controlled trials: part 17 of a series on evaluation of scientific publications. Dtsch Arztebl Int.

[21] Flecha OD, Douglas de Oliveira DW, Marques LS, Gonçalves PF (2016). A commentary on randomized clinical trials: how to produce them with a good level of evidence. Perspect Clin Res.

[22] Biswas A, Castanov V, Li Z, Perlas A, Kruisselbrink R, Agur A, Chan V (2018). Serratus plane block: a cadaveric study to evaluate optimal injectate spread. Reg Anesth Pain Med.

[23] Mayes J, Davison E, Panahi P, Patten D, Eljelani F, Womack J, Varma M (2016). An anatomical evaluation of the serratus anterior plane block. Anaesthesia.

[24] Kunigo T, Murouchi T, Yamamoto S, Yamakage M (2018). Spread of injectate in ultrasound-guided serratus plane block: a cadaveric study. JA Clin Rep.

[25] Hotta K (2019). Fascial plane blocks: anatomical structures that affect the spread of local anesthetic. J Anesth.

[26] Murad MH, Asi N, Alsawas M, Alahdab F (2016). New evidence pyramid. Evid Based Med.

[27] Camacho FC, Segura-Grau E (2019). Continuous serratus anterior plane block provides analgesia in multiple rib fractures: a case report (Article in Portuguese). Braz J Anesthesiol.

[28] Kunhabdulla NP, Agarwal A, Gaur A, Gautam SK, Gupta R, Agarwal A (2014). Serratus anterior plane block for multiple rib fractures. Pain Physician.

[29] Bossolasco M, Bernardi E, Fenoglio LM (2017). Continuous serratus plane block in a patient with multiple rib fractures. J Clin Anesth.

[30] Lin J, Hoffman T, Badashova K, Motov S, Haines L (2020). Serratus anterior plane block in the emergency department: a case series. Clin Pract Cases Emerg Med.

[31] Fu P, Weyker PD, Webb CA (2017). Case report of serratus plane catheter for pain management in a patient with multiple rib fractures and an inferior scapular fracture. A A Case Rep.

[32] Rose P, Ramlogan R, Sullivan T, Lui A (2019). Serratus anterior plane blocks provide opioid-sparing analgesia in patients with isolated posterior rib fractures: a case series. Can J Anaesth.

[33] Durant E, Dixon B, Luftig J, Mantuani D, Herring A (2017). Ultrasound-guided serratus plane block for ED rib fracture pain control. Am J Emerg Med.

[34] Hernandez N, de Haan J, Clendeninn D (2019). Impact of serratus plane block on pain scores and incentive spirometry volumes after chest trauma. Local Reg Anesth.

[35] Martel ML, Robidoux MR, Morris JL, Reardon RF (2020). Feasibility and initial experience with continuous nerve blocks by emergency physicians. Am J Emerg Med.

[36] Martinez T, Belveyre T, Lopez A (2020). Serratus plane block is effective for pain control in patients with blunt chest trauma: a case series. Pain Pract.

[37] McLean J, Cooke S, Burns B, Reid C (2019). First reported helicopter in-flight serratus plane block for rib fractures. Air Med J.

[38] Rose P, Ramlogan R, Madden S, Lui A (2019). Serratus anterior plane block home catheter for posterior rib fractures and flail chest. Can J Anaesth.

